# COVID-19 Disease and Outcomes among Critically Ill Patients: The Case of Medical Nutritional Therapy

**DOI:** 10.3390/nu14071416

**Published:** 2022-03-29

**Authors:** Dimitrios Karayiannis, Sotirios Kakavas, Zoi Bouloubasi, Zafeiria Mastora

**Affiliations:** 1 Department of Clinical Nutrition, “Evangelismos” General Hospital of Athens, Ypsilantou 45-47, 10676 Athens, Greece; zoippp@yahoo.com; 2 Intensive Care Unit, Center for Respiratory Failure, “Sotiria” General Hospital of Chest Diseases, 152 Mesogeion Avenue, 11527 Athens, Greece; sotikaka@yahoo.com; 3 First Department of Critical Care Medicine and Pulmonary Services, Evangelismos General Hospital, National and Kapodistrian University of Athens, 11527 Athens, Greece; zafimast@yahoo.gr

The recent COVID-19 pandemic, which resulted from SARS CoV-2 coronavirus infection, contributed toa rapid increasein hospital and intensive care unit (ICU) admissions [1]. Although during the last 3 years there have been numerous research publications on patient care, data concerning the role of the dietary approach in the overall treatment of the disease are minimal. Moreover, with regard to the dietary approach during COVID-19 critical illness, practice guidelines are based on data which were developed too quickly and were basedontargeted recommendations on feeding the critically ill [2]. Since then, new sources of data have emerged, which clearly display significant nutritional challenges.

In relation to the organization of the provision of nutritional care, data from the USA proclaim that only about 1 in 5 centers (21%) have developed a specialized protocol for the provision of nutritional treatment [3]. Among these centers, some of the key issues raised by feeding staff included the difficulty of feeding awake patients and the reluctance to add additional PN in cases of inadequate EN administration. The results of this study reported that the emphasis given tothe exclusive administration of food through the gastric tract was rarely effective in this group of patients.

Why are we so interested in providing individualized nutritional therapy to critically ill patients with COVID-19? Firstly, these patients tend to exhibit a significantly higher length of hospital stay compared to other subcategories of critically ill patients, while simultaneously they will need nutritional support for longer periods, according to the data of Arrieta et al., as presented in the current Special Issue (SI) [4]. In addition, according to recent findings, about 8 in 10 patients will leave the ICU at high nutritional and sarcopenia risk [5]. This finding supports the hypothesis that these patients obviously do not receive optimal nutritional care. Additionally, disease symptoms and de-arrangement of the nutritional status persists until 6 months after hospital discharge, as an interesting French study published in this SI informed us [6]. The authors supported the hypothesis that COVID-19 patients had a much higher risk of developing muscle weakness, malnutrition and functional loss. On the other hand, the presence of obesity or overweight seems to be positively related to the likelihood of death during hospitalization among COVID-19 patients [7].

In relation to the energy requirements of the critically ill COVID-19 patients, studies report that there are substantial differences compared to other groups of critically ill patients, characterized by a prolonged hypermetabolic state [8]. The hyperinflammatory response during COVID-19 disease is a systemic phenomenon leading to a cytokine storm with an increase in systemic markers such as C-reactive protein (CRP) and erythrocyte sedimentation rate (ESR) [9]. This prolonged period of hyperinflammation causes several metabolic disorders and extremely high levels of energy expenditure, and secondarily other changes, such as severe insulin resistance, tumor overload and hypernatremia secondary to imperceptible loss and osmotic diuresis [10]. Patients usually present with hypocalcaemia, hyperkalaemia or hypokalaemia, hyperphosphataemia (secondary to muscle breakdown and mitochondrial insufficiency), and hypertriglyceridaemia.

Are there any data on best nutrition support practices? Unfortunately, there are no data from randomized clinical trials, with the exception of some micronutrient supplementation studies with immunomodulating actions [11]. Although reduced levels of vitamin C, D, Zinc and selenium may be considered as a risk factor for patients with COVID-19, data from published studies so far highlight the lack of well-designed clinical intervention, evaluating the effect of the administration of either individual nutrients or their combination [11]. Kakavas et al., in the present SI hypothesized that immunonutrient administration could be associated with a reduction in the de novo synthesis and/or release of histamine and other mast cell mediators that could mediate, at least in part, the immune and microvascular alterations present in COVID-19 [12].

When it comes to the feeding route, retrospective data from 176 patients suggest that the majority of patients tend to reach protein and caloric requirements through the use of Parenteral Nutrition during the first week of hospitalization [13]. One recent prospective monocentric study conducted in a large Greek ICU and published in this SI evaluated the influence of feeding practices on patient outcomes. In this study, the route for delivery of full nutritional support (enteral vs. parenteral nutrition) during the second week of hospitalization was not associated with mortality risk [14]. However, there was a significant difference in length of hospital stay and duration of mechanical ventilation support, both being lower in the enteral feeding group.In relation to the macronutrient composition of the diet, fragmentary reports show a tendency for lower mortality in those who receive adequate amounts of protein, but these data do not come from high-quality studies [15]. Regarding more critical questions, such as the optimal macronutrient ratio, when to use parenteral nutrition, when to start enteral nutrition in patients with vasopressors, how tofeed prone ventilated patients, how to consider extubation phase and whether there is utility tousing special feeds (fish oil, immunonutrition), there are choices, but no answersyet.

In conclusion, the key concept identified in this SI was that optimizing dietary practices for patients both during their ICU stay and beyond is crucial. Clinicians should be capable of managing their patients both during their hospitalization and rehabilitation phase, in order to confirm the continuous care and to minimize the susceptibility of adverse events due to malnutrition.
